# Behaviour Real-Time Spatial Tracking Identification (BeRSTID) used for Cat Behaviour Monitoring in an Animal Shelter

**DOI:** 10.1038/s41598-022-22167-3

**Published:** 2022-10-20

**Authors:** B. H. Eagan, B. Eagan, A. Protopopova

**Affiliations:** 1 Animal Welfare Program, Faculty of Land and Food Systems, University of British Columbia, Vancouver, Canada; 2 Independent Researcher, Toronto, Canada

**Keywords:** Image processing, Behavioural methods

## Abstract

Efficiently tracking animal behaviour in an animal shelter has direct lifesaving applications. Individualized care and early recognition of distress in cats are often missed. However, monitoring behaviour is a challenge as time and financial resources are often limited, and the size and needs of animal populations within shelters are commonly in flux. Our research required a method of behavioural observation that was simple, accessible, used limited human and computer resources and allowed for real-time feedback. Here, we present BeRSTID, an open-source behaviour real-time spatial tracking identification system demonstrated on six cats in an animal shelter using unique 2D fiducial markers. The markers were attached to custom veterinary paper identification collars for feedback on individual animal behaviour over time. Our findings show that BeRSTID correlated closely to human-coded data in both real-time and post-event processing modes of eating and drinking behaviours of cats in naturalistic shelter environments. By building upon a lateral concept of marker tracking for direct applied use in a new context, we present a low-barrier user-friendly solution using common technologies that can track animals for research and, with further development, may help improve welfare in animal care facilities such as shelters. Extensions of BeRSTID may be generalized to track unique subjects in varied environments for multiple use cases.

## Introduction

Monitoring behaviour is essential to providing care for animals as well as in animal behaviour and welfare research^1,2^. Currently, recording animal behaviour is primarily conducted via methods of observation, either live or through video recording^2^. In-person observation may introduce the risk of observer effects and impact animal behaviour^2^. Whereas monitoring behaviour through video eliminates this risk, behavioural video recording and coding remains time-intensive^2^ with limitations due to human fatigue^3^. Video review is also inherently delayed, diminishing the ability to intervene in cases of animal distress. Systems that allow remote real-time monitoring of animal behaviour show promise for animal behaviour research and use in animal care settings.

Animal behaviour recording is particularly critical in animal care facilities such as shelters, which house an estimated 6.6 million cats and dogs annually in Canada and the United States alone^4,5^. While animal capacity varies across shelters, one shelter facility may house and care for hundreds of cats in an average month^6^. Detecting slight behaviour changes shows promise for informing standards of care for lifesaving interventions in shelters^1^. Standards of care highlight the importance that animals receive individual care, including daily health and behavioural evaluations, to detect potential welfare problems^1^. Cats are the most commonly housed animal in shelters and often experience high levels of in-shelter stress^7,8^.

Detailed behaviour tracking in shelters is vital for improving cat health and welfare^1^. Understanding if a cat is showing critical maintenance behaviours, such as eating and drinking, especially within the first few days in a shelter, can provide insight into how they cope in confinement^9^. Individual care and early detection of issues help inform intervention when needed and avoid animals languishing in the shelter^1^. If an animal is showing signs of not coping in a shelter, interventions such as moving to a quieter room or foster home if available, beginning a behavioural treatment program^10^, or starting anxiety-reducing medication^11^ may be beneficial. However, animal shelters are commonly overwhelmed with animal intake and, with limited resources, often face substantial challenges in the daily assessment and management of animal welfare^6^. Behaviour recording of animals in shelters is usually limited to brief in-person assessments and daily cage inspection*.* As a result, detailed and consistent behavioural data critical for detecting early signs that a cat is not coping well in shelter, or those required for research purposes, may be missed.

Tracking a specific cat in scenarios when animals are housed in pairs and groups becomes increasingly challenging, especially if individual cats cannot be reliably identified, or resource use such as eating or drinking cannot be monitored by cage inspection. In addition to cases when cats need to be kept isolated to prevent infectious disease spread, cats may be kept isolated in shelters to simplify behaviour tracking, due to the identified difficulty of tracking behaviour when housed communally^1^. Some cats who benefit from group housing (e.g., cats from a group environment accustomed to stable social interactions) may be prevented from group-housing access due to observation needs presenting an animal welfare concern^1^.

Advancements in technology are increasingly incorporated into animal behaviour and welfare research and care, including radio-frequency identification (RFID) tags used for collecting spatial information on animal behaviour^12^. However, common barriers of RFID use in shelters include cost, number of RFID readers required for regions of interest^13^ and potential difficulties operating near metal surfaces^14^ (a common material in animal shelters for sanitation purposes). Artificial intelligence (AI) can be incorporated to offer insights into animal behaviour and welfare, and several studies have used advanced AI applications to inform behavioural research^15–21^. AI for tracking individual behaviour is increasingly used for owned companion animals (e.g., Mundell et al.^22^); however, the use of AI in animal shelters is scarce. While individual tracking systems may benefit shelters, these approaches often involve preparing a training dataset of the subjects, training an inference model, and prioritizing pose-estimation over specific animal identification (e.g., TRex by Walter & Couzin)^19^. Therefore, while advanced AI methods show extensive research potential, many approaches are not currently feasible for implementation in shelter environments.

This paper introduces an alternate computer-vision based BeRSTID, an open-source behaviour real-time spatial tracking identification system, demonstrated on six cats in an animal shelter setting. Tracking is done by attaching a unique machine-readable 2D tag to custom veterinary paper identification collars, significantly simplifying the task of tracking individual animals. Barcode assisted detection has been shown in insects^23–27^ and songbirds^3^. While some concern is that the attachment of artificial markers or tags can result in behavioural interference^19,28^*,* animal shelters already commonly require veterinary identification collars for manual identification. The extension of a barcode detection method to monitor collar-wearing animal behaviour in real-time has not been previously explored.

## Results

### Marker tracking system for automatic cat behaviour monitoring in an animal shelter

We deployed the computer vision marker tracking system (BeRSTID) on six cats (*Felis catus*) in an animal shelter. We found that BeRSTID highly correlated to videos behaviourally coded by a single naïve human observer (HO) of cat eating and drinking behaviour in a shelter for both post-event processing and real-time monitoring (Table 1). Data collection occurred in fully operational animal shelters. Therefore, this system was tested in a real-world setting, and encountered issues likely to occur if deploying this for animal care or research purposes. Consequently, we have used these issues to inform best practices, and method refinements throughout.

**Table 1:** Intraclass correlation coefficients (ICC) for observer agreement between a human observer (HO) recorded behaviour and computer vision marker detection output.

Recording type	Cats (n)	Duration monitoring eating (HH:MM)	Duration monitoring drinking (HH:MM)	Eating BeRSTID and HO ICC	Food ROI presence BeRSTID and HO ICC	Drinking BeRSTID and HO ICC	Water ROI presence BeRSTID and HO ICC
Post-event (by frame)	2	31:37	31:37	0.77	0.80	0.55	0.62
Post-event with correct resource placement (by frame)	2	31:37	17:22	0.77	0.80	0.92	0.96
Real-time monitoring (by second)	4	40:00	40:00	0.75	NA	0.85	NA

Region of interest (ROI) presence indicates detection or HO recorded presence within a defined ROI. All p values < 0.001.

**Figure 1:**
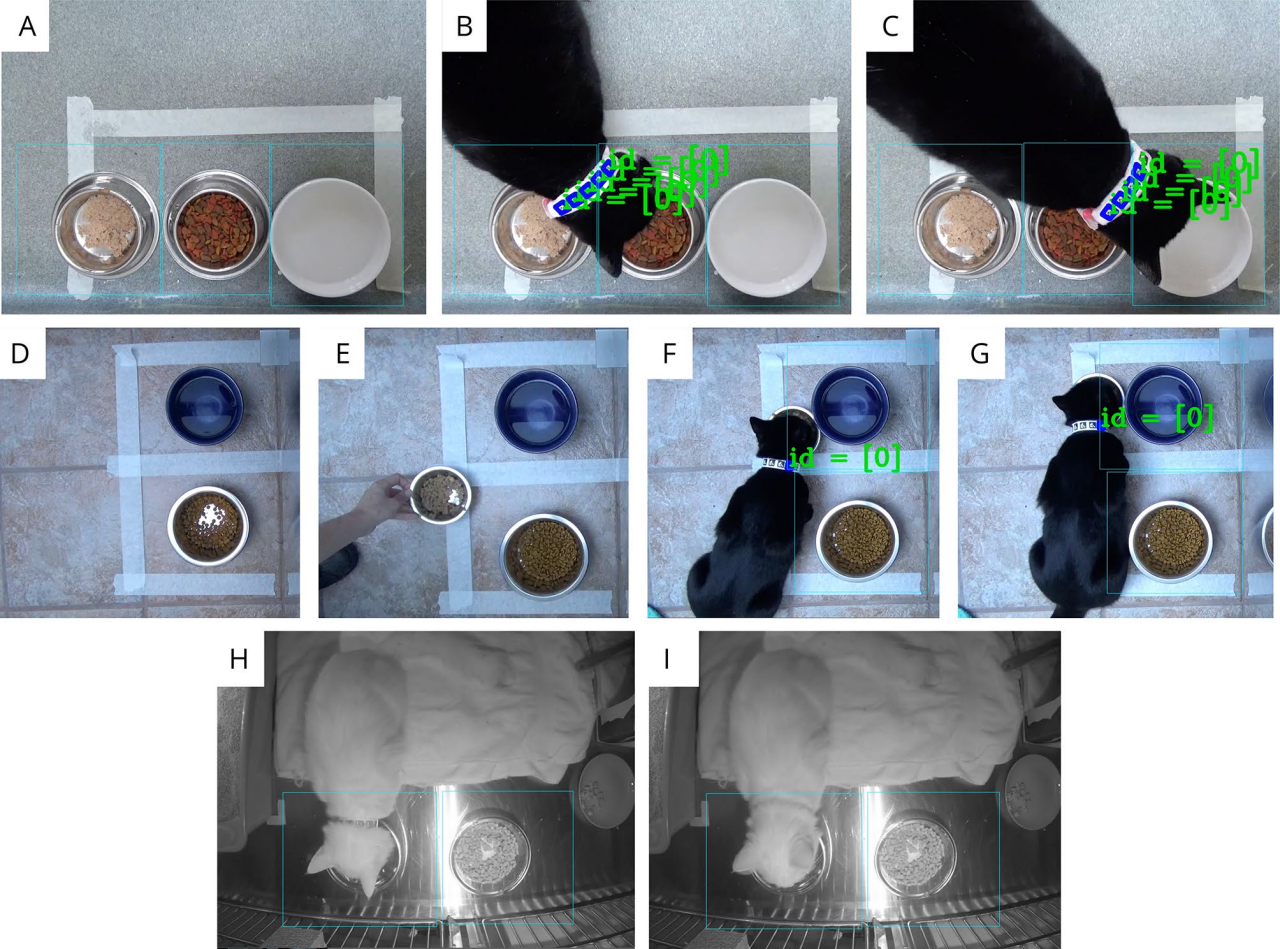
(**A**) Example of resources placed too close to one another. (**B**) Example of a detection occurring in region of interest (ROI) 0 [wet food] simultaneously while the cat is eating and being detected in ROI 1 [dry food] due to too close resource placement. (**C**) Example of a detection occurring in ROI 1 [dry food] simultaneously while the cat is drinking and being detected in ROI 2 [water] due to too close resource placement. (**D**) Example of resource placement including two ROIs (ROI 0 [water] ROI 1 [dry food]) with adequate spacing to ensure ROIs are not too close. (**E**) Example of an issue of an additional unintended resource being added within the ROI of ROI 0 [water] ROI 1 [dry food]. (**F**) Example of the use of an additional resource of wet food added to ROI region of (ROI 0 [water] ROI 1 [dry food]); therefore, detection represented in the green text occurred within the ROI of the unused resource. (**G**) Movement of the additional resource by a cat due to lightweight metal bowl as the cat moved the unintended additional resource across the ROIs. (**H**) An example of a cat using a resource in ROI 0 [water] and due to fur coverage, a detection was missed. (**I**) An example of a cat occupying a space within an ROI but not using the resource, resulting in no detection.

As multiple staff and volunteers from the British Columbia Society for the Prevention of Cruelty to Animals (BC SPCA) continuously cared for cats in the shelter environments, unsurprisingly, the primary error encountered was incorrect resource placement (Fig. 1A–G). Further, due to power issues in the shelter environment, 31:37 (HH:MM) out of 48:00 of video data were available for running through the marker tracking system algorithm for post-event analysis, and 17:22 out of the total were available including proper resource placement for the water bowl.

Cat collars remained intact and detectable by the BeRSTID algorithm for the maximum duration of validation monitoring (48:00). Cats were observed for signs of discomfort or distress with wearing a paper veterinary identification (ID) collar by BC SPCA staff, and as none were observed, no cats involved in validation required collar removal.

The BeRSTID tracking system showed a high degree of agreement with behaviour manually coded by the HO. Intraclass Correlation Coefficients (ICC)^29,30^ computed between the BeRSTID output and HO coded behaviour are summarized in Table 1. ICC between region of interest (ROI) presence and behaviour within the ROI had high correlation for both eating (kappa = 0.86, p < 0.001) and drinking (kappa = 0.91, p < 0.001), showing that when the cat is visible in the ROI space around the resource, there is a high likelihood they are using that resource. ICC calculation between the HO and a second human observer, HO2, showed high agreement between observers for identifying cat presence in the food ROI (kappa = 0.94, p < 0.001) and eating behaviour (kappa = 1.0, p =  < 0.001).

### Method deployment & best practices for increasing accuracy

**Figure 2:**
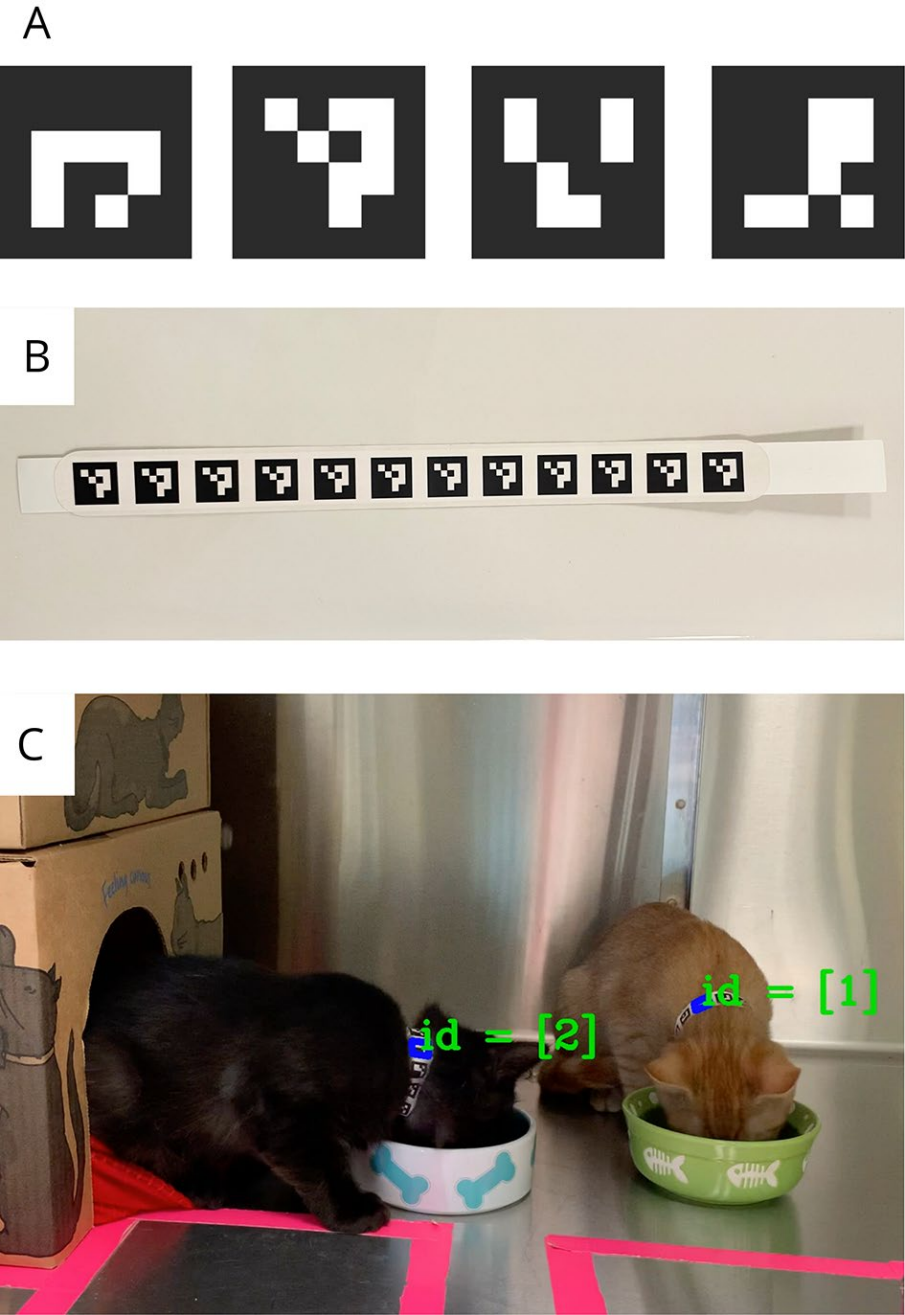
(**A**) Examples of individual 16-bit ArUco markers of unique markers for individual identification numbers of 0, 1, 2 and 3. (**B**) TabBand paper veterinary ID collar with a custom sticker, including repeating ID marker. (**C**) Two cats with unique collar markers. Detection of each ID is visible in annotated output as indicated by “id = [1]” and “id = [2]”, showing individual cat IDs detected in the video.

**Figure 3:**
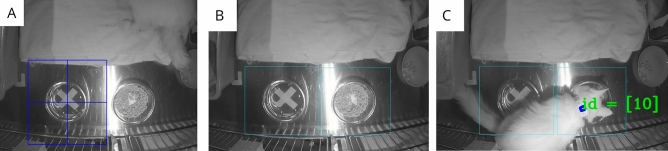
(**A**) A region of interest (ROI) is manually drawn on the video output. (**B**) Two regions of interest are drawn around resources of interest, including a water and food bowl. (**C**) A marker for ID 10 is detected in the food bowl ROI.

During deployment, three common human caused problems arose for detection regarding resource placement, specifically (1) placement of resources too close to one another (Fig. 1A–C), (2) placement of additional resources within a specified ROI (Fig. 1E), and (3) placement of resources in a lightweight bowl that may be moved by the cat while being used (Fig. 1F,G). These encountered issues present simple solutions to inform method refinement and best practices.

Figure 1A–C demonstrates an example of a resource placement issue, with the placement of resources too close to one another resulting in problems associating detection to resource usage. Placement of a water bowl, dry food bowl, and wet food bowl closely touching required ROIs to be drawn very near to one another (Fig. 1A). As a result, when the cat was using a resource (Fig. 1B and C), detections occurred in both the ROI of the behaviour being observed and the neighbouring ROI of the unused resource. Therefore, the resources must be placed at an adequate distance from one another such that detection in a nearby ROI will not occur erroneously (Fig. 1D). Resource placement issues were also found to arise if an unintended resource was placed within an ROI (Fig. 1E). As a result, when using the added unintended resource, detections occurred within incorrect ROIs (Fig. 1F,G). Therefore, unintended resources must not be placed within ROIs for accurate monitoring.

Resource placement and maintenance are critical for the BeRSTID system to reliably detect eating and drinking behaviour in cats. Proper order and maintenance of the ROIs was found to improve accuracy, as evidenced by improved correlation with human observation (Table 1). While proper placement and maintenance of resources can improve the method’s detection, and high accuracy with human observer recordings was observed, some aspects of using this method in a real-world setting on animals presented some limitations (Fig. 1H,I). Firstly, when testing with cats with longer hair, some instances showed a long coat blocking the code, preventing consistent detection (Fig. 1H). As the BeRSTID system required the entire ID to be visible, including a white outline surrounding the ID marker, hair coverage resulted in some missed detections. While this occasionally occurred, as demonstrated by the high agreement with human-coded videos, this issue appeared infrequently.

Further, as the ROI is drawn and stationary, if a cat occupies the space within the ROI but does not use the resource (Fig. 1I), this can decrease accuracy when validating against the human observation. This is a particular consideration if recording cats in small spaces, where the likelihood of occupying an area of the ROI is higher due to an overall low amount of space, when group-housing or communally housing cats in larger rooms, the likelihood of them occupying an area within an ROI but not using the resource is likely lower. While sniffing behaviour was observed, due to the limited occurrences and very brief durations, as evidenced by high correlation with eating and drinking behaviour (Table 1), sniffing did not result in low agreement with human-observed resource use.

## Discussion

Here, we present the use of a computer vision marker tracking system, BeRSTID, on six cats in a naturalistic animal shelter setting. This method demonstrated a low complexity, open-source, accessible and inexpensive manner for collecting behaviour data, intended to require minimal technical skill. Current automatic animal behaviour monitoring systems, such as RFID^13^, marker tracking^3^ and markerless tracking using AI^19^ all demonstrate advanced applications for animal research. However, these solutions are not currently feasible in a shelter environment due primarily to cost, and complexity of implementation. BeRSTID presents a lower barrier of entry in terms of price and complexity. Using unique 2D fiducial markers attached to custom veterinary paper identification collars, BeRSTID presents an opportunity for real-time feedback on individual cat eating and drinking behaviour over time that is highly correlated to videos behaviourally coded by a HO. Considering that this degree of detail in manual monitoring is not feasible in an animal shelter, this shows that BeRSTID likely extends current manual observation methods by allowing for continuous, recorded monitoring even overnight in an unobtrusive way. By building upon a lateral idea of tracking for direct applied use in a new context, we demonstrate effective and efficient animal behaviour tracking using this BeRSTID system. While presented here in cats, this method shows promise for use for any collar-wearing species requiring simple and continuous spatial identification of individual animals in complex environments. However, further validation is needed before it can be applied in additional contexts.

This method presents promise for accessible animal shelter research and direct animal care applications by having functionality in both post-event processing and real-time behaviour monitoring. As the implementation is written using Python 3, this solution is supported on ARM, × 86, and M1 CPU architectures. The computing power required was also minimal, enabling real-time detection even with low powered Raspberry Pi 3 computers, which are low cost and readily available for under CAD 50 (www.canakit.com). The BeRSTID system does not depend on expensive high-resolution cameras, showing successful tracking with low-cost USB webcams and Raspberry Pi camera modules. These factors combined lead to an affordable, portable, and accessible vision-based monitoring solution.

When implementing BeRSTID, resource placement must be carefully considered, as the logged data output will only be as accurate as the placement of the resources. To that end, refinements and future applications may include introducing ROI shape capabilities beyond a rectangle shape and ID markers for the resources to define resource location further and serve as a system health check to ensure detections are continuously occurring. Additionally, unique ID markers could be added to the resources, with an ROI boundary relative to the marker position rather than statically fixed in the camera field of view. This may indicate a hardware failure or changes to the environment itself. This would also serve as a health check on the vision system, providing the opportunity to alert when resources are not visible. With an understanding of the range of potential ROI issues, we expect careful vetting of resource placement, and a redundant power supply would dramatically improve our coverage. Based on this information, we predict 90–100% coverage to be obtainable.

This method and dataset also present future directions in machine learning (ML) and automated animal behaviour monitoring. A notable challenge in ML vision solutions focused on recognizing a specific subject is having a dataset available to facilitate recognizing a particular subject. This method provides a mechanism to generate labelled ML training data, identifying unique animals in many lighting conditions and angles and identifying likely interaction with resources. This ML could, for example, increase the accuracy of the behavioural prediction in cases where a marker appears in two ROIs. It could also be used as a redundant unique identification method, detecting such instances as a long-coated cat whose marker is obscured.

Further, these models could be trained using past data and validated continuously with incoming data. This could be conducted using BeRSTID for several days and training deep learning solutions such as a Convolutional Neural Network (CNN) simultaneously based on past data. Validation would be done against real-time data and evaluated against the BeRSTID data. If the ML inferences agree with BeRSTID predictions, the system could switch to the ML-powered solution (perhaps no longer requiring markers) or a combination of both^31^.

In the rapidly advancing animal behaviour tracking technology field, the BeRSTID system demonstrates a simple solution, aiming for ease of implementation in a wide range of animal care and research settings. However, further refinements to the BeRSTID system are needed before any technician can readily introduce it for care into real-world environments such as an animal shelter. To be helpful in an animal care setting, the BeRSTID system should be set up within a room to allow the system to run continuously whenever needed. This approach would require careful planning of resource placement to ensure the maximum number of resources could be monitored with minimal cameras while maintaining some distance between resources to avoid competition and to allow for sanitization procedures between populations. Careful planning of resource placement could allow for the system to remain installed within a room, and monitor new populations as they enter the shelter. Further, while the BeRSTID post-event processing method required a delay in information, the real-time monitoring method allowed for immediate behavioural updates to be viewed by the research team in OneDrive. However, this required the research team to view and interpret the data to share with the shelter staff. Simple extensions of this technology to automate the outputs to easily interpretable updates, such as daily or hourly summaries of behaviours automatically viewable by staff, would make this technology immediately more interpretable in a practical setting.

This technology's continual growth and extension could greatly benefit its practicality in an animal care setting. Our objective is for this open-source and accessible system to be used, further developed, and validated in other room configurations, animal care and research settings, and for varied species, and behaviours. We aim for this study to further showcase an animal shelter setting as a practical testing ground for experimenting with non-invasive automated technologies and applications, such as BeRSTID. Due to an animal shelter's extensive and ever-changing population of animals, shelters allow for a wide range of data collection opportunities. Further, creating and testing technology in an animal shelter may not only benefit development but may help provide much-needed information on animal behaviour and welfare in a resource-limited environment that could help inform lifesaving intervention.

## Methods

### Ethics approval

This study was approved by the University of British Columbia’s Animal Care Committee (ACC) (A20-0295), and all methods were performed in accordance with the ACC policies and guidelines. All methods and results are reported in accordance with the ARRIVE Guidelines^32^.

### BeRSTID tracking system for automatic cat behaviour monitoring in an animal shelter

The fiducial marker system BeRSTID developed for cat behaviour monitoring consisted of four components automatically tracking eating and drinking behaviour. The BeRSTID system included (1) a veterinary paper identification (ID) collar fitted to a cat with a unique marker visible, (2) a video input device, (3) a computational device running the cross-platform detection software, and (4) physical regions of interest (ROI) for resources to denote specific behaviours (e.g., a food or water bowl). This system was tested on two use cases (1) pre-recorded videos for post-event video processing (i.e., for analyzing stored video data), and (2) real-time processing for real-time monitoring (i.e., for immediate feedback with potential for animal care applications).

The video data was passed frame by frame to our specialized BeRSTID software (Supplementary Methods 1). When ID(s) were detected in the frame, each ID’s location, timestamp, and ROI presence were logged. The BeRSTID system was employed to test marker detection for monitoring cat behaviour in a shelter setting. The focus behaviours were eating and drinking, selected due to the critical nature of observing these behaviours in animal care and when researching these animals, and the importance of early detection of the absence of these behaviours in an animal shelter for health and welfare purposes^9^.

Data collection occurred on cats that entered the British Columbia Society for the Prevention of Cruelty to Animals (BC SPCA), opportunistically selected by BC SPCA staff. Cats were unsystematically selected based on availability within the shelter population, requiring only that they did not have any skin conditions in the neck region and were, therefore, able to wear paper veterinary ID collars. Due to public health limitations for the COVID 19 pandemic, BC SPCA staff attached the collars with individual IDs assigned for individual cats, and installed study cameras under remote instruction by the study team. Following BC SPCA animal care guidelines, staff assessed cats wearing collars daily for signs of irritation with collar wearing and removed collars if cats were showing signs of discomfort.

#### Post-event recording

Post-event recording and processing of the eating and drinking behaviour of two singly-housed adult domestic short-haired cats was conducted in April 2021 at the Vancouver BC SPCA. Two Sony Handycams (HDR-CX405) were placed within each cat housing room, approximately 1.5 m above food and water bowls. These cameras recorded cats for 48:00 (HH:MM), and video monitoring began as soon as the cats entered the room. The video streams saved to an SD card were then subjected to the marker detection code that captured individual IDs and timestamps within defined regions of interest, including (1) at the food bowl and (2) at the water bowl. For post-event processing, the BeRSTID program was run using a command-line interface on a Macbook Pro equipped with an Intel i7-9750H, and 16 GB of RAM. In the post-event analysis workflow, an option was initially employed to output annotated video (Supplementary Video 1) complete with ROI and ID number layered onto the output for setup and early validation. A comma-separated value (CSV) output file was also generated for post-event analysis. The output file included every frame, which contains a detected ID from the fiducial dictionary and logs the ID number, x and y coordinates, ROI presence, timestamp, and frame count. In post-processing, the software guarantees every frame is analyzed.

#### Real-time monitoring

Real-time monitoring was conducted on four adult domestic longhair group-housed cats in November 2021 at the Richmond BC SPCA. One Raspberry Pi computer with an Arducam 1080P Day & Night Vision USB Camera Module (Arducam) was placed approximately 0.5 m above two communal food and water resources for the group-housed cats. Monitoring occurred continuously in both resource locations within the group-housed room for 48:00.

For real-time monitoring, a real-time video was passed through BeRSTID, with logged data continuously uploaded to OneDrive cloud computing storage and stored locally in CSV format. In this mode, video data was streamed in real-time to the BeRSTID algorithm. In this capture mode, the user may choose to output annotated video, as well as CSV data or CSV data alone. When monitoring real-time, the temporal priority superseded the processing of every frame. Thus, frames could be dropped if computer resources are exceeded. In our 48:00 of Raspberry Pi 3 analysis, however, a frame rate of 30fps was sustained. As the real-time system alone did not store video output, while not a requirement for method deployment, for validation against human observer (HO), a Nest Indoor camera was placed beside the Arducam for later behaviour coding.

#### BeRSTID system validation against human observers

Following completion of data collection, videos from post-event recording and the Nest camera (used only for real-time validation) were manually coded continuously by a single trained HO to determine the daily occurrences, the timestamp, and duration of drinking and eating behaviours (Supplementary Data 1, Supplementary Data 2). The ethogram used for behaviour coding is included in Table 2. For the initial validation conducted using post-event recording, both the presence of the cat within the ROI, and the behaviour of the cat (eating, drinking, or sniffing) was recorded. This was to assess if the presence of the cat detected within an ROI was a reliable indicator of an animal using the resource within the ROI. For real-time monitoring, only eating and drinking behaviour was behaviourally coded by the HO.

**Table 2:** Ethogram for behaviour coding of post-event processing and real-time video monitoring of eating, drinking, sniffing, and region of interest (ROI) presence.

Behaviour	Behaviour determination	Region of interest (ROI) determination
Eating	Cat actively ingests food or other edible substances from the bowl by means of chewing with the teeth and swallowing. It can be identified through audible chewing/eating sounds and visible head movements (nodding, or raising and lowering the head). Eating is only observed after lowering the head to the food bowl and ingesting food. This does not include exploring, investigating, sniffing, standing over, not engaging in eating, or approaching the food bowl. It is considered a separate eating event if the cat has not been involved in the above-detailed behaviours for around 1 min (60 s or 1800 frames)	Any section of the cat’s head from neck up is present within the defined region of interest
Drinking	Cat actively ingests water or other liquids from the bowl by lapping up with the tongue. It can be identified through audible lapping sounds and visible head movements (nodding, or raising and lowering the head). Drinking is only observed after lowering the head to the water bowl and ingesting liquid. It does not include exploring, investigating, sniffing, standing over, or approaching the water bowl. It is considered a separate drinking event if the cat has not engaged in any above-detailed behaviours for over 1 min (60 s or 1800 frames)	Any section of the cat’s head from neck up is present within the defined region of interest
Sniffing	Cat lowers head (nose) within two inches of food or water bowl or litter but does not engage in eating or drinking behaviour	NA

Note: Presence within ROI not recorded for real time processing.

Post-event processing was coded frame by frame by a HO for the presence or absence of food ROI, water ROI, eating, drinking, and sniffing (for a total duration of 31:37). Real-time monitoring was coded second by second by a HO for the presence or absence of eating, drinking and sniffing behaviour (for a total duration of 40:00). Human-coded results were compared to BeRSTID output, to assess correlation between HO and BeRSTID recorded timestamp and duration of each recorded behaviour. An ICC^29,30^ was calculated in R Studio (RStudio 2022.02.3 + 492)^33^ between BeRSTID output and human-coded behaviour to assess if the BeRSTID system provided a reliable approximation of cat behaviour compared to manual human observation. For post-event analysis, ICC was calculated between HO and BeRSTID output for food ROI, water ROI, eating and drinking. An ICC was also calculated between HO food ROI and HO eating, and HO water ROI and HO drinking to assess if presence of a cat’s head in the ROI of a resource correlated with actively using that resource. For real-time analysis, ICC was calculated for eating and drinking behavior and BeRSTID output. Further, sniffing behaviour was not included in correlation analysis, due to the rare occurrences and short durations of this behaviour. To assess observer reliability of human-coded behaviour, a second naïve human observer (HO2) coded 3:00 of stored video, and an ICC was calculated between the primary HO and HO2.

#### Marker creation and detection of collars

The visual fiducial markers were a core component of this method. Markers were printed and placed on specific cat collars expected to enter the field of view of the video sensors. Using the BeRSTID system, when markers were detected within a field of view, each marker ID’s location, timestamp, and ROI presence were logged. The custom markers used were created using the ArUco module of the OpenCV library as described in OpenCV Detection of ArUco Markers^34^*.* Several geometric configurations are supported by ArUco based on the aspect ratio of the 2D surface available and the number of unique subjects. A set of IDs are referred to as a dictionary, and all subjects must have IDs generated from a common dictionary^35^.

A dictionary of 16 individual 16-bit markers with a square 1:1 aspect ratio was created (Fig. 2A). These were individually printed on collars, with one ID repeatedly displayed sequentially on a single collar (Fig. 2B). To be detected, a marker must be visible to the camera, including a white edge visible surrounding the marker. The computer system will not identify the marker if the white edge is not visible. For specific marker detection methods, see Garrido-Jurado et al.^35^.

With the dictionary of fiducial markers generated, a challenge remains in transferring these IDs to the physical media attached to the cat subjects as collars. To achieve this, 25 cm × 2.5 cm custom stickers were repeatedly printed with a single ID across a single sticker. Then, stickers were attached to a standard TabBand paper veterinary identification collar (Fig. 2B). Individual marker IDs on collars allow individual cats with different collars to be detectable in video output (Fig. 2C), and the repeating code allows for multiple opportunities for detection depending on the angle of the cat’s neck.

#### Defining regions of interest

Marker detection within a specified ROI can be conducted with BeRSTID. By specifying a region within the video frame of interest, BeRSTID looked for markers only within the specified section. To include an ROI, the region must first be drawn (Fig. 3A), including capabilities for several ROIs (Fig. 3B). Once ROIs are drawn and video processing begins, detection of individual markers within an ROI begins (Fig. 3C), and the output specifies which ROI was included in the detection. ROIs were constrained to square bounding boxes in the initial implementation, encouraging perpendicular camera placement above the resources such as a water dish.

## Supplementary Information

Supplementary Information 1.

Supplementary Information 2.

Supplementary Information 3.

Supplementary Video 1.

Supplementary Legends.

## Data Availability

All data generated or analysed during this study are included in this published article (and its Supplementary Information files).
