# Supplementary material for: Behaviour Real-Time Spatial Tracking Identification (BeRSTID) used for Cat Behaviour Monitoring in an Animal Shelter
Source: Sci Rep. 2022 Oct 20;12:17585. doi: 10.1038/s41598-022-22167-3 (PMC9584257; doi:10.1038/s41598-022-22167-3)
Supplement: Supplementary file 2 — Supplementary Information 2. [file 41598_2022_22167_MOESM2_ESM.docx]

**Supplementary Data 2**

Video files for validation available at <https://drive.google.com/drive/folders/1hI3loTEhqvi2S3gsfuINO5zfAdJyuupA?usp=sharing>.
