# Supplementary material for: Behaviour Real-Time Spatial Tracking Identification (BeRSTID) used for Cat Behaviour Monitoring in an Animal Shelter
Source: Sci Rep. 2022 Oct 20;12:17585. doi: 10.1038/s41598-022-22167-3 (PMC9584257; doi:10.1038/s41598-022-22167-3)
Supplement: Supplementary file 3 — Supplementary Information 3. [file 41598_2022_22167_MOESM3_ESM.docx]

**Supplementary Methods 1**

BeRSTID code available at <https://github.com/benrules2/BeRST>.
