# Supplementary material for: Behaviour Real-Time Spatial Tracking Identification (BeRSTID) used for Cat Behaviour Monitoring in an Animal Shelter
Source: Sci Rep. 2022 Oct 20;12:17585. doi: 10.1038/s41598-022-22167-3 (PMC9584257; doi:10.1038/s41598-022-22167-3)
Supplement: Supplementary file 5 — Supplementary Legends. [file 41598_2022_22167_MOESM5_ESM.docx]

# Supplementary Materials

Supplementary Methods 1: BeRSTID Code

Supplementary Data 1: CSV Files for Correlation Calculation

Supplementary Data 2: Videos Files for Validation

Supplementary Video 1: Annotated Video Output Example
